# An Expanded Gene Catalog of Mouse Gut Metagenomes

**DOI:** 10.1128/mSphere.01119-20

**Published:** 2021-02-24

**Authors:** Jiahui Zhu, Huahui Ren, Huanzi Zhong, Xiaoping Li, Yuanqiang Zou, Mo Han, Minli Li, Lise Madsen, Karsten Kristiansen, Liang Xiao

**Affiliations:** a State Key Laboratory of Bioelectronics, School of Biological Science and Medical Engineering, Southeast University, Nanjing, China; b BGI-Shenzhen, Shenzhen, China; c Laboratory of Genomics and Molecular Biomedicine, Department of Biology, University of Copenhagen, Copenhagen, Denmark; d Institute of Marine Research, Bergen, Norway; e Qingdao-Europe Advanced Institute for Life Sciences, BGI-Shenzhen, Qingdao, China; f Shenzhen Engineering Laboratory of Detection and Intervention of Human Intestinal Microbiome, BGI-Shenzhen, Shenzhen, China; g BGI College & Henan Institute of Medical and Pharmaceutical Science, Zhengzhou University, Zhengzhou, China; University of California, Davis

**Keywords:** diet, gene catalog, metagenomic species, mouse gut metagenome

## Abstract

High-quality and comprehensive reference gene catalogs are essential for metagenomic research. The rather low diversity of samples used to construct existing catalogs of the mouse gut metagenome limits the numbers of identified genes in existing catalogs. We therefore established an expanded catalog of genes in the mouse gut metagenome (EMGC) containing >5.8 million genes by integrating 88 newly sequenced samples, 86 mouse gut-related bacterial genomes, and 3 existing gene catalogs. EMGC increases the number of nonredundant genes by more than 1 million genes compared to the so-far most extensive catalog. More than 60% of the genes in EMGC were assigned to *Bacteria*, with 54.20% being assigned to a phylum and 35.33% to a genus, while 30.39% were annotated at the KEGG orthology level. Nine hundred two metagenomic species (MGS) assigned to 122 taxa are identified based on the EMGC. The EMGC-based analysis of samples from groups of mice originating from different animal providers, housing laboratories, and genetic strains substantiated that diet is a major contributor to differences in composition and functional potential of the gut microbiota irrespective of differences in environment and genetic background. We envisage that EMGC will serve as a valuable reference data set for future metagenomic studies in mice.

**IMPORTANCE** We established an expanded gene catalog of the mouse gut metagenome not only to increase the sample size compared to that in existing catalogs but also to provide a more comprehensive reference data set of the mouse gut microbiome for bioinformatic analysis. The expanded gene catalog comprises more than 5.8 million unique genes, as well as a wide range of taxonomic and functional information. Particularly, the analysis of metagenomic species with the expanded gene catalog reveals a great novelty of mouse gut-inhabiting microbial species. We envisage that the expanded gene catalog of the mouse gut metagenome will serve as a valuable bioinformatic resource for future gut metagenomic studies in mice.

## INTRODUCTION

Mice are among the most widely used animal models for biomedical studies to decipher the complex interplay between the gut microbiota and host phenotypes (1–4). Amplicon sequencing of the 16S rRNA gene has been widely used for analyses of the gut microbiota due to low costs and short analysis cycles. However, the taxonomic information is, in most cases, limited to the genus level, and amplicon sequencing generally provides limited information on function (5, 6). A key to the use of mouse models for detailed functional analyses of the gut microbiota is the availability of comprehensive catalogs of microbial genes and derived metagenomic species (MGSs)/metagenome-assembled genomes (MAGs). The first catalog of genes in the mouse gut microbiome included 2.6 million nonredundant genes from fecal samples of 184 mice (7). Subsequent studies further explored the diversity and functional potential of the mouse gut microbiota by isolating and sequencing an increasing number of bacterial strains from the mouse gut (8–11) and establishing a mouse intestinal bacterial collection (miBC), depositing bacterial strains and associated genomes from the mouse gut (9). Recently, Lesker et al. generated an integrated mouse gut metagenome catalog (iMGMC), comprising 4.6 million unique genes and 830 high-quality MAGs, and by linking MAGs to reconstructed 16S rRNA gene sequences, they provided a pipeline enabling improved prediction of functional potentials based on 16S rRNA gene amplicon sequencing (12).

Here, we constructed an expanded mouse gut metagenome catalog (EMGC) by integrating 3 published gene catalogs, including the gene catalog of the mouse gut metagenome (MGGC) released in 2015 comprising 2,571,074 genes (7), a feed and diet gene catalog for mice (FDGC) (13), the integrated mouse gut metagenome catalog (iMGMC) (12), 72 available sequenced mouse gut-related bacterial genomes (8–11), 14 high-quality genomes assembled from published sequencing data of isolates (9), and 88 newly shotgun-sequenced samples. Our new nonredundant reference gene catalog comprises 5,862,027 genes and was annotated by NR (released on 5 January 2019) and KEGG (release 87) databases (14). Finally, we generated 902 MGSs from the gene abundance profiles for 326 laboratory mice of EMGC and compared these MGSs with the high-quality MAG collection (12) and the recent collection of bacteria isolated from the mouse gut (11). By combining these individual data sets, we increased the number of sequenced bacterial genes of the mouse gut microbiome by more than 1 million and significantly increased the mapping ratio of reads obtained by shotgun sequencing of samples from the mouse gut and fecal samples, providing a resource for future studies on the mouse gut microbiota.

(This article was submitted to an online preprint archive [15].)

## RESULTS

### Construction and evaluation of EMGC.

**FIG 1:**
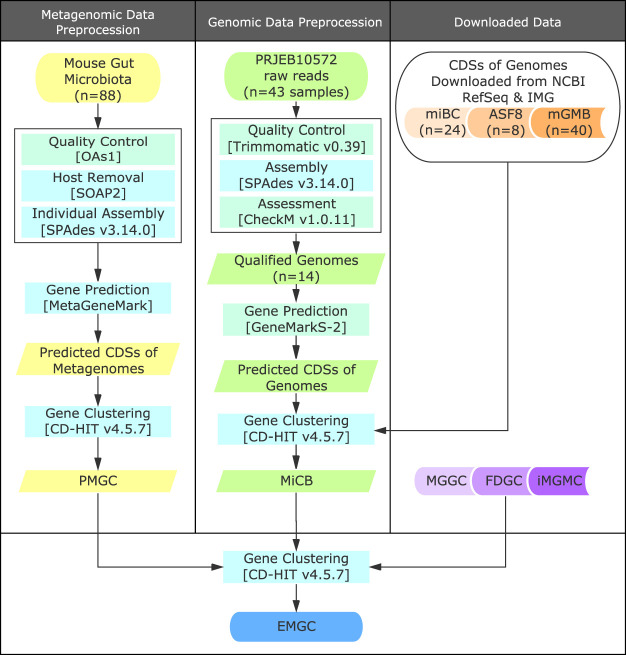
Construction of the EMGC. Metagenomic sequencing data of 88 mouse gut metagenomes were processed by the pipeline as displayed to generate nonredundant genes for PMGC. Unassembled strains of miBC (under BioProject PRJEB10572) were assembled and filtered by genome quality (completeness, >90%; contamination, <5%) of assembled genomes. Qualified genomes were used for gene prediction. CDSs from assembled genomes and downloaded genomes were gathered and clustered to MiCB. PMGC and MiCB along with 3 downloaded gene sets, FDGC, MGGC, and iMGMC, were merged to generate EMGC.

Fecal samples from 88 C57BL/6J male mice were sequenced using the BGISEQ-500 platform providing 1,098-Gb high-quality host-free data with an average of 12.47 Gb per sample (see Table S1A in the supplemental material) and a catalog comprising 2,602,584 nonredundant genes (PMGC). We next used 72 mouse gut-related bacterial genomes (8–11) from IMG and NCBI RefSeq and 14 high-quality genomes (completeness >90% and contamination <5%) assembled from reads accessible from PRJEB10572 (9) (Table S1B) to generate a mouse gut cultured bacterial gene set (MiCB). All gene catalogs, including MGGC (7) together with FDGC (13) and iMGMC (12), downloaded from GigaDB and the Zenodo repository (Table S1C), respectively, were integrated to construct an expanded nonredundant mouse gut bacterial gene catalog (EMGC) (Fig. 1). The expanded catalog comprises 5,862,027 genes, which is more than twice the number of genes in the MGGC (7) and 1 million genes more than the iMGMC catalog (12) (Table 1). Thus, 18.93% of the genes in EMGC are not represented in either the iMGMC or MGGC (see Fig. S1).

**TABLE 1:** General features of gene catalogs

Catalog	Sample size (*n*)	Total no. of ORFs^*a*^	Length (bp)				*N*_50_	*N*_90_
			Total	Avg	Max	Min		
PMGC^*b*^	88	2,602,584	1,920,079,578	737.76	120,489	102	981	396
MGGC^*c*^	184	2,572,074	1,959,483,705	761.83	120,489	102	1,014	408
FDGC^*d*^	54	793,847	585,096,360	737.04	23,610	102	978	396
MiCB^*e*^	NA	267,801	251,087,538	937.59	79,287	100	1,206	504
iMGMC^*f*^	292	4,499,720	3,505,479,714	779.04	120,399	102	1,107	390
EMGC^*g*^	434	5,862,027	4,542,473,508	774.90	120,489	100	1,104	393

a ORFs, open reading frames.

b PMGC, a sub-mouse gut gene catalog from 88 mouse gut metagenomes.

c MGGC, gene catalog of mouse gut metagenome released in 2015.

d FDGC, feed and diet gene catalog for mice.

e MiCB, mouse intestinal cultured bacteria gene set.

f iMGMC, integrated mouse gut metagenome catalog.

g EMGC, an expanded gene catalog of mouse gut metagenomes.

10.1128/mSphere.01119-20.1 FIG S1 Unique and shared genes between MGGC, iMGMC, and EMGC. Download FIG S1, PDF file, 0.1 MB. Copyright © 2021 Zhu et al. 2021 Zhu et al. https://creativecommons.org/licenses/by/4.0/ This content is distributed under the terms of the Creative Commons Attribution 4.0 International license.

10.1128/mSphere.01119-20.10 TABLE S1 (A) Statistics for sequencing data of the 88 samples from this study. (B) Selection for 86 mouse gut-related sequenced prokaryotic genomes. (C) Data resources used in this study. (D) Background information and the subgroups of all 326 samples. (E) Mapping rates of 40 mouse fecal metagenomes. (F) Mapping rates of 34 mouse cecal metagenomes. (G) Comparison of coverage of KO pathway between MGGC, iMGMC, and EMGC. (H) Comparison of genus relative abundance (>1e−5) between HF and LF diets. (I) Comparison of KO relative abundance (>1e−5) between HF and LF diets. (J) Information of MGSs with more than 700 genes presented in EMGC. Download Table S1, XLSX file, 0.3 MB. Copyright © 2021 Zhu et al. 2021 Zhu et al. https://creativecommons.org/licenses/by/4.0/ This content is distributed under the terms of the Creative Commons Attribution 4.0 International license.

**FIG 2:**
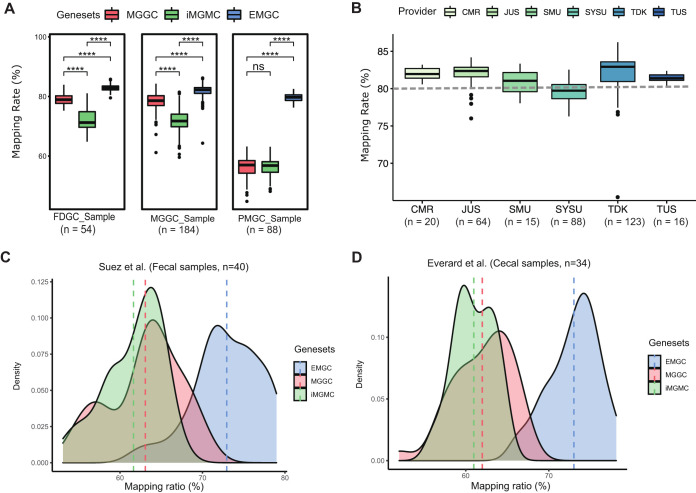
Performance of the EMGC. (A) Comparison of mapping rates between MGGC, iMGMC, and EMGC. ****, BH-adjusted *P* value of <0.0001 by Wilcox rank sum test. (B) Display of mapping rates among samples’ providers, including the Wallenberg Laboratory for Cardiovascular and Metabolic Research (CMR), the Jackson Laboratory in the United States (JUS), the laboratory animal center of Southern Medical University (SMU), the laboratory animal center of Sun Yat-Sen University (SYSU), and Taconic in Denmark (TDK) and in the United States (TUS). Dashed line represents a mapping rate of 80%. (C and D) Density curves for the mapping rates of fecal metagenomes and cecal metagenomes which were not included in gene catalog construction. Dashed lines represented the average values of the mapping rate of each gene catalog.

To compare the performance of EMGC with that of MGGC and iMGMC, we mapped sequencing reads from the FDGC, MGGC, and PMGC studies to the three catalogs. Of the sequencing reads from PMGC, which is part of EMGC, 55.72% were mapped to MGGC and 56.56% to the iMGMC. In contrast, the EMGC allowed mapping of 79.52% of the reads (Fig. 2A), close to the maximum achievable mapping rate in prokaryotes (16).

A comparison of mapping rates of reads from 326 fecal samples obtained from different mouse strains and providers (Table S1D) to those from EMGC demonstrated that mice from the laboratory animal center at Sun Yat-Sen University exhibited a lower mapping rate than samples from the other providers, where the median mapping rates were higher than 80% (Fig. 2B). Median mapping rates of reads obtained from all mouse strains were also higher than 80% (see Fig. S2A). Richness estimated by Chao2 indicated that our EMGC covered 98.24% of the genes in the 326 fecal samples (Fig. S2B), whereas the incidence-based coverage estimator (ICE) suggested that 97.49% of the genes were covered.

10.1128/mSphere.01119-20.2 FIG S2 Evaluation of EMGC in mapping. (A) Mapping rates based on the EMGC grouped by mouse strains. Dotted line indicates a mapping rate of 80%. (B) Rarefaction curve of Chao2 index and unique gene count. Download FIG S2, PDF file, 0.3 MB. Copyright © 2021 Zhu et al. 2021 Zhu et al. https://creativecommons.org/licenses/by/4.0/ This content is distributed under the terms of the Creative Commons Attribution 4.0 International license.

To further evaluate the quality of the EMGC, we mapped the metagenomic data obtained from 40 fecal samples from control mice and mice that had consumed noncaloric artificial sweeteners (17) and metagenomic data obtained from 34 cecal samples from control mice and mice treated with prebiotic (18) (Table S1C). For the reads obtained in the study of Suez et al. (17), 63.07% mapped to the MGGC and 61.04% to iMGMC, whereas 72.94% mapped to the EMGC (Fig. 2C; Table S1E). For the reads obtained from the study by Everard et al. (18), 61.99% mapped to MGGC and 60.97% mapped to iMGMC, but 73.01% of the reads mapped to the EMGC (Fig. 2D; Table S1F). Together, these results demonstrate a significantly increased mapping rate of reads using the EMGC as a reference.

### Taxonomic and functional characteristics of EMGC.

We taxonomically annotated the genes of EMGC using Kaiju (19) and the NCBI NR database to provide an overview of the taxonomical composition visualized by a Krona plot (20). This plot revealed that 67% of the genes were able to be annotated (see Fig. S3). We assigned 54.20% of the genes to the phylum level and 44.30% of the genes to the family level (see Fig. S4A and B). We next annotated the genes in the EMGC to the KEGG (release 87) database (14) and identified 6,704 KEGG functional orthologs (KO) and 290 KEGG pathways (see Fig. S5).

10.1128/mSphere.01119-20.3 FIG S3 General view of the taxonomic composition of EMGC in a Krona pie chart. Download FIG S3, PDF file, 0.1 MB. Copyright © 2021 Zhu et al. 2021 Zhu et al. https://creativecommons.org/licenses/by/4.0/ This content is distributed under the terms of the Creative Commons Attribution 4.0 International license.

10.1128/mSphere.01119-20.4 FIG S4 Stacked bar plots showing the distributions of the taxonomic annotations of the EMGC at the phylum (A) and family (B) levels. Download FIG S4, PDF file, 0.1 MB. Copyright © 2021 Zhu et al. 2021 Zhu et al. https://creativecommons.org/licenses/by/4.0/ This content is distributed under the terms of the Creative Commons Attribution 4.0 International license.

10.1128/mSphere.01119-20.5 FIG S5 Distribution of annotated functional pathways according to KEGG in EMGC. Download FIG S5, PDF file, 0.01 MB. Copyright © 2021 Zhu et al. 2021 Zhu et al. https://creativecommons.org/licenses/by/4.0/ This content is distributed under the terms of the Creative Commons Attribution 4.0 International license.

**FIG 3:**
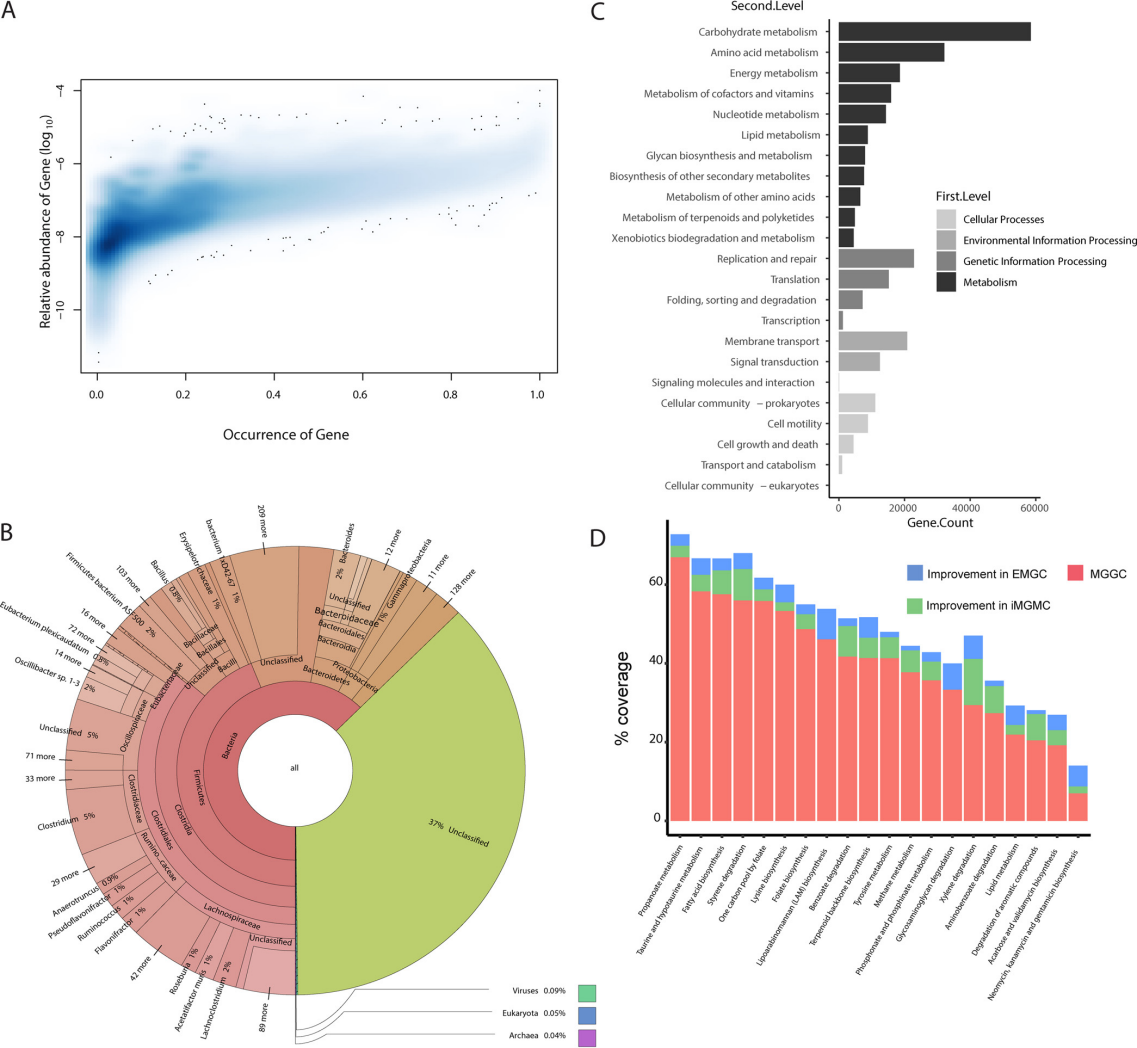
Description of new genes included in EMGC. (A) Two-dimensional (2D) density histogram showing the distribution of occurrences and mean relative abundances of new genes. (B) General display of taxonomic composition of new genes by Krona. (C) Frequency of functional pathways associated with the new genes. (D) Stacked histogram of KO coverage of functional pathways improved in EMGC compared to that in MGGC and iMGMC. Coverage is calculated as [(annotated KO numbers)/(total KO numbers)] × 100 in a given pathways.

To further examine the quality of the EMGC, we calculated the occurrence frequency and average abundance of the 1,109,381 genes not present in the previous MGGC and iMGMC. As shown in Fig. 3A, 40.41% of these genes exhibited an occurrence frequency and mean abundance higher than 0.1 and 10^−8^, respectively. We also extracted taxonomic and functional information of these new genes. Annotation of the new genes at the species level revealed that the top 5 species could be assigned to *Oscillibacter* sp. 1-3, *Firmicutes* bacterium ASF500, Acetatifactor muris, bacterium 1xD42-67, and Eubacterium plexicaudatum (Fig. 3B), all isolated from the mouse gut based on information from the NCBI BioSample database. In relation to functions, the general distribution of KEGG pathways in these additional genes is similar to the overall distribution in EMGC (Fig. 3C). We identified 189 KOs in EMGC which are not present in either MGGC or iMGMC. Furthermore, 42 KEGG pathways are covered by additional KOs (Table S1G) in EMGC. For 5 KEGG pathways, including lipoarabinomannan (LAM) biosynthesis, glycosaminoglycan degradation, xylene degradation, neomycin, kanamycin and gentamicin biosynthesis, and terpenoid backbone biosynthesis, we found that more than 5% of the additional KOs are only represented in EMGC compared to that in iMGMC (Fig. 3D; Table S1G).

### Changes in the microbiota composition and functional potential.

**FIG 4:**
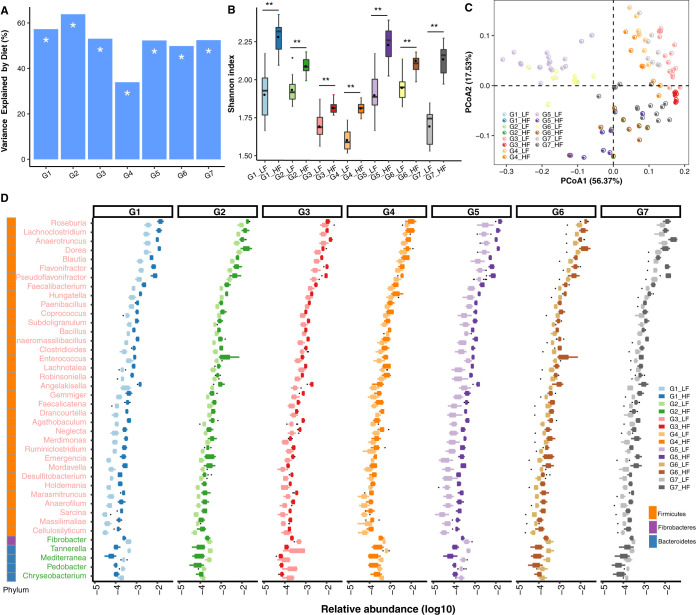
Influence of diet on the composition of the microbiota. (A) PERMANOVA to estimate the influence of diet on the composition of gut metagenomes among all 7 sample groups. G1, C57BL/6 mice provided by Taconic in Denmark (TDK) and hosted in National Institute of Nutrition and Seafood Research of Norway (NIFES); G2, Sv129 mice provided by TDK and hosted in NIFES; G3, C57BL/6 mice provided by the Jackson Laboratory in the United States (JUS) and hosted by Pfizer-I; G4, C57BL/6 mice provided by Taconic in the United States (TUS) and hosted in Pfizer-I; G5, C57BL/6 mice provided by TDK and hosted in the University of Copenhagen (KU); G6, Sv129 mice provided by TDK and hosted in KU; G7, C57BL/6 mice provided by the laboratory animal center of Sun Yat-Sen University (SYSU). *, *P* < 0.05. Shannon index (**, BH-adjusted *P* < 0.01, Wilcox rank sum test) (B) and PCoA based on genus profile (C) for 7 groups fed the HF and LF diets. (D) Genera differently enriched (BH-adjusted *P* < 0.05, Wilcox rank sum test; relative abundance, >1e−5) in mice fed the HF and LF diets among all 7 groups. Genera in light red represent genera enriched in HF-diet-fed mice, while genera in light green represent genera enriched in LF-diet-fed mice.

We reported earlier that the mouse gut metagenome is affected by animal providers and housing as well as strain and diet (7). To investigate to what extent diet affected the gut metagenomes independently of strain and providers, we selected 7 groups (G1 to G7) of samples from different strains from different providers fed a low-fat (LF) diet or a high-fat (HF) diet and housed in the same facility (see details in Table S1D). We estimated the impact of diet on the variation of gut microbiota based on the relative abundance profiles of genera and KOs using a permutational multivariate analysis of variance (PERMANOVA). The analyses indicated that diet explained at least 33.9% (*P* value = 0.003) of the total variation at the genus level and 47.3% (*P* value = 0.006) at the KO level (Fig. 4A; see also Fig. S6A). Compared to that for mice fed an LF diet, mice fed an HF diet exhibited an increase in alpha diversity at the genus level independent of housing laboratories, strains, and providers (Fig. 4B). In contrast, at the KO level, alpha diversity in mice fed an LF diet generally, except for group 7, exhibited an increased diversity compared to that for mice fed an HF diet (Fig. S6B). Principal-coordinate analysis (PCoA) similarly confirmed that the diet strongly influenced the genus profile (Fig. 4C) and the KO profile (Fig. S6C).

10.1128/mSphere.01119-20.6 FIG S6 Influence of diet on the composition of bacterial functions (KOs) based on EMGC. (A) PERMANOVA estimates of the influence of diet on gene functions in the gut metagenomes of different groups. G1, C57BL/6 mice provided by Taconic in Denmark (TDK) and hosted in the National Institute of Nutrition and Seafood Research of Norway (NIFES); G2, Sv129 mice provided by TDK and hosted in NIFES; G3, C57BL/6 mice provided by the Jackson Laboratory in the United States (JUS) and hosted by Pfizer-I; G4, C57BL/6 mice provided by Taconic in the United States (TUS) and hosted in Pfizer-I; G5, C57BL/6 mice provided by TDK and hosted in the University of Copenhagen (KU); G6, Sv129 mice provided by TDK and hosted in KU; G7, C57BL/6 mice provided by the laboratory animal center of Sun Yat-Sen University (SYSU). *, *P* < 0.05. (B) Boxplot for of the Shannon indexes of KOs in mice fed HF and LF diets in each group. **, BH-adjusted *P* < 0.01; *, BH-adjusted *P* < 0.05, Wilcox rank sum test. (C) PCoA for the KO profile. Download FIG S6, PDF file, 0.3 MB. Copyright © 2021 Zhu et al. 2021 Zhu et al. https://creativecommons.org/licenses/by/4.0/ This content is distributed under the terms of the Creative Commons Attribution 4.0 International license.

To further examine diet-induced changes, we examined genera and KOs enriched in samples from either HF- or LF-diet-fed mice by Wilcoxon rank sum test. As shown in Fig. 4D, in all 7 groups, the 36 genera found at higher abundance in samples from HF-diet-fed mice belong to *Firmicutes*, whereas four genera within the *Bacteroidetes* phylum and one genus within the *Fibrobacteres* phylum were found at higher abundance in samples from LF-diet-fed mice (Fig. 4D; Table S1H). However, whereas 363 KOs were enriched in LF-diet-fed mice, only 270 KOs were detected at higher abundance in HF-diet-fed than in LF-diet-fed mice (Table S1I). To investigate which taxa contributed to the disparate response to LF and HF diet at the taxonomy and the functional levels, we identified the taxa at the phylum level that contributed to the enrichment of KOs. Whereas genera within the *Bacteroidetes* phylum were the main contributor accounting for 6.02% of the KOs enriched in LF-diet-fed mice, genera within the *Firmicutes* phylum accounted for 3.96% of the KOs enriched in HF-diet-fed mice (see Fig. S7).

10.1128/mSphere.01119-20.7 FIG S7 Cumulative relative abundances of top 10 phyla contributing to genes related to HF diet-enriched KOs and LF diet-enriched KOs. Download FIG S7, PDF file, 0.1 MB. Copyright © 2021 Zhu et al. 2021 Zhu et al. https://creativecommons.org/licenses/by/4.0/ This content is distributed under the terms of the Creative Commons Attribution 4.0 International license.

### Construction of metagenomic species.

We identified 902 metagenomic species (MGSs; >700 genes) using the relative gene abundances based on 326 fecal samples obtained from different mouse strains and providers using MGS canopy clustering and taxonomic annotation as described previously (21). The 902 MGSs were assigned to 122 taxa (see Fig. S8; Table S1J). We also generated MGS profiles for the 7 groups of mice fed an LF or an HF diet. The Shannon indices and PCoA plot revealed a clear effect of diet, independent of mouse strain and provider (see Fig. S9A and B).

10.1128/mSphere.01119-20.8 FIG S8 Cladogram with bars for the taxonomic distribution of MGSs clustered from EMGC gene profiles. Download FIG S8, PDF file, 0.1 MB. Copyright © 2021 Zhu et al. 2021 Zhu et al. https://creativecommons.org/licenses/by/4.0/ This content is distributed under the terms of the Creative Commons Attribution 4.0 International license.

10.1128/mSphere.01119-20.9 FIG S9 Influence of diet on the composition of MGSs based on EMGC. (A) Boxplot showing how different diets in each group affect the Shannon index based on MGS profiles. **, BH-adjusted *P* < 0.01, Wilcox rank sum test. (B) PCoA for MGS profile of HF and LF diets in the 7 sample groups. Download FIG S9, PDF file, 0.5 MB. Copyright © 2021 Zhu et al. 2021 Zhu et al. https://creativecommons.org/licenses/by/4.0/ This content is distributed under the terms of the Creative Commons Attribution 4.0 International license.

**FIG 5:**
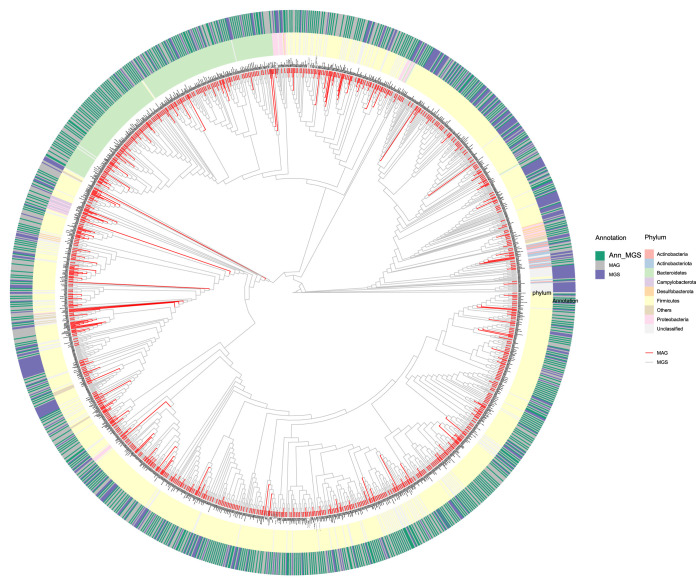
Phylogenetic tree of the 902 MGSs and 830 high-quality iMGMC MAGs. MUMi distances for MGSs and MAGs were used to construct the phylogenetic tree using hierarchical clustering. MAGs are shown as red branches and MGSs as gray branches. The outer ring shows the relation between MGSs and MAGs. MGSs which have a MUMi value of >0.54 are marked as “Ann_MGS” in green blocks, otherwise, in purple blocks. MAGs are all in gray blocks. Colored blocks in the inner cycle indicate phyla assigned to MGSs and MAGs.

We next compared the 902 MGSs with the 830 high-quality nonredundant MAGs generated in the iMGMC project (12) and 115 bacterial genomes from the mouse gut microbial biobank (mGMB) project (11). Five hundred fifty-nine (61.97%) MGSs were classified as the same species as the MAGs from the iMGMC project (maximal unique match index [MUMi] value [22–24] >0.54) (Table S1J). As shown in Fig. 5, *Firmicutes* and Bacteroides were the most prevalent phyla among all MGSs and MAGs. We also identified 56 MGSs representing genomes of species from the mGMB project, and of these, 8 MGSs could be identified as mGMB genomes, but not as MAGs (Table S1J). Of note, for more than one-third of the MGSs, we were unable to identify corresponding entities in the MAG collection or in the cultured genomes collection.

## DISCUSSION

The EMGC represents the most comprehensive catalog of genes in the mouse gut microbiome. It covers samples from feces and cecum from different mouse strains fed different diets, obtained from different providers, and housed in different laboratories. The majority of the genes identified in this study were assigned to known species, which might improve the coverage of known species and the detection of low-abundant taxa. The improvement in KO coverages of a number of pathways will enhance the functional characterization of the mouse gut microbiota. In addition, the analysis of samples from different mouse strains from different animal providers and different housing laboratories confirms the pronounced effect of diets on the taxonomic and functional composition of the gut microbiota.

In spite of the increased number of genes in EMGC, there are still some limitations. The sample size and variation of sample types in the EMGC are still small. The majority of samples included in EMGC were collected from C57BL/6 mice, which might affect the applicability in studies on other laboratory mouse strains and wild-caught mice. Many confounding factors in addition to those addressed in the present study will most probably impinge on the gut microbiota (1, 3, 4, 25). It therefore seems important to include more samples from other mouse strains to gain further insights into the effect of confounding factors, which may lead to pronounced variability in the mouse gut microbiota, which again might limit the reproducibility of biomedical research using mouse models (25, 26). Although both culture-independent and culture-dependent studies on the mouse gut microbiota have been carried out to improve the understanding of host-microbe interaction in mouse models, the majority of the mouse gut metagenome members still remain relatively uncharacterized (7, 9, 11, 12). Besides, we also noticed the limitations of the construction approach of EMGC. EMGC is based on metagenome assembly and focused on the gene-level characterization of the mouse gut microbiome. The applications of protein-level metagenome assembler (27), annotation (28), and binning tools (12, 29–31) are needed in future studies to enhance our understanding of the composition and functions of the mouse gut microbiota.

Even though more and more metagenomic analysis methods are being developed at an increasing speed, gene catalogs are still necessary resources, not only to provide reliable and consistent taxonomic annotation but also to reduce the gap between phylogenetic and functional biases (16, 32). The high-quality reference gene catalog, EMGC, together with the 902 metagenomic species, is able to support and improve accurate metagenome-wide association analyses using mouse models, which may assist in functional characterization of observed correlations between the microbiota composition and the functional potential in relation to host phenotypes.

## MATERIALS AND METHODS

### Data acquisition.

DNA from 88 stool samples of C57BL/6J wild-type male mice, collected from the laboratory of BGI-Wuhan, was extracted and shotgun sequenced using the BGISEQ-500 platform and paired-end 100-bp (PE100) sequencing as described previously (33). An optimized sequencing quality filter for the cPAS-based BGISEQ platform, OAs1 (33), was applied in the quality control step, followed by host removal with SOAP2 (v2.21, parameters: -m 0 -x 1000 -c 0.9) (34) using GRCm38 (https://www.ncbi.nlm.nih.gov/assembly/GCF_000001635.20/) as the reference mouse genome. Individual assembly of metagenomic reads was performed using metaSPAdes v3.14.0 (parameters: -k 49; other parameters were set to the default) (35, 36).

Genes were predicted by MetaGeneMark (v2.7) (37) from metagenome-assembled contigs with a length of >500 bp and filtered by length of >100 bp. Redundant predicted genes were removed by CD-HIT (v4.5.7, parameters: -G 0 -n 8 -aS 0.9 -c 0.95 -d 0 -r 1 -g 1) (38) in order to generate a sub-mouse gut gene catalog (PMGC).

Raw reads of 43 unassembled bacterial genomes from the miBC (EBI project identifier [ID] PRJEB10572) were downloaded from EBI and filtered by Trimmomatic (v 0.39) (39). Draft genomes were assembled separately by SPAdes (-k 29,39,49,69 –careful) (40, 41) and filtered by CheckM (v1.0.13) (42). After assessment using the criteria of completeness of >90% and contamination of <5%, the remaining 14 genomes were used for gene prediction by GeneMarkS-2 (v1.07) (43).

A total of 72 genomes, including 24 sequenced strains of miBC (9), 8 genomes of the altered Schaedler flora (8) (PRJNA175999 to PRJNA176003, PRJNA213740, PRJNA213743), and 40 genomes of mGMB (11) (released before 26 February 2019, PRJNA486904), as well as their coding sequences (CDSs) and translated CDSs were all downloaded from the NCBI RefSeq database and the Integrated Microbial Genomes (IMG) database (44). We gathered CDSs from 86 bacterial genomes and filtered out genes smaller than 100 bp. We clustered CDSs using CD-HIT (v4.5.7, parameters: -G 0 -n 8 -aS 0.9 -c 0.95 -d 0 -r 1 -g 1) (38), establishing a gene catalog termed mouse intestinal cultured bacteria gene set (MiCB). Detailed information on the included genomes is provided in Table S1B in the supplemental material.

All public mouse-related microbial metagenomic data sets used in this study are listed in Table S1C, including (i) 184 host-free sequenced mouse gut microbiomes and the gene catalog of mouse gut metagenome (MGGC) (7), (ii) 54 mouse gut microbiomes and the related gene catalog (FDGC) (13), (iii) 830 high-quality dereplicated MAGs and the iMGMC gene catalog (12), (iv) 40 mouse fecal metagenomes (17), and (v) 34 mouse cecum metagenomes (18).

### Construction of EMGC and selection of new genes.

All downloaded genes were filtered by length of >100 bp and integrated to construct the EMGC using CD-HIT (v4.5.7, parameters: -G 0 -n 8 -aS 0.9 -c 0.95 -d 0 -r 1 -g 1) (38). The output of EMGC clusters was analyzed to generate a list for new genes in EMGC that are not present in iMGMC and MGGC. Metagenomes were mapped to gene catalogs by SOAP2 (v2.21, parameters: -m 0 -x 1000 -c 0.95) (34). Mapping rates between groups and catalogs were compared by Wilcoxon rank sum test (R ggpubr package). *P* values were adjusted by using the Benjamini-Hochberg method. The profile of relative gene abundances for the 326 laboratory mice (see Table S1D for an overview of these mice) was calculated based on the method of Qin et al. (45) using EMGC. Richness estimation by the Chao2 index and incidence-based coverage estimator (ICE) was calculated based on the gene abundance profiles (16). The occurrence and average abundance of new genes were calculated using the relative gene abundance profiles.

### Taxonomic and functional annotation.

Genes predicted from metagenome assemblies were taxonomically annotated by Kaiju (v1.6.3) (19) using the NCBI-NR database (released on 5 January 2019) and the parameters of the program were set to “-a greedy -e 5 -E 0.01 -v -z 4 -s 65.” For genes from mouse gut-related bacterial genomes, we kept the original taxonomic information of the genomes and assigned them to the corresponding genes. All genes were searched against KEGG (version 87) (14) by DIAMOND blastx mode (v2.0.6.144, parameter: –evalue 0.001) (46) for functional annotation. The filtering parameters of DIAMOND were set with a query coverage threshold of 80% and a minimum score of 60 (16, 47). The best hits which met the above-described criteria were retained. DIAMOND results were turned into functional annotation based on the information provided by KEGG to create a gene KO list for the generation of KO relative abundance profiles. The taxonomic and functional information of new genes of EMGC was extracted for further analysis.

### Evaluation of the effect of diet on the gut microbiota.

To evaluate the consistent effect of diet among providers and mouse strains on taxonomic and functional composition of gut metagenomes, samples from 7 groups fed high-fat (HF) or low-fat (LF) diets and representing different providers and mouse strains were selected (Table S1D). The calculation of relative abundance profiles for taxa and KO was according to Qin et al. (45). Permutational multivariate analysis of variance (PERMANOVA) (R vegan package) based on Bray-Curtis dissimilarity was applied to determine the influence of diet on gut metagenomes within different groups. The Shannon index of the relative abundance profiles was used to estimate alpha diversity of the samples. Principal-coordinates analysis (PCoA) of selected samples was performed based on the relative abundance profiles using Bray-Curtis dissimilarity (R ape4 package) to visualize the effect of diet on the bacterial composition of the gut microbiota. The Wilcox rank sum test was used for analysis of differences of genera and KO relative abundance profiles. *P* value adjustment was applied for multiple hypothesis testing using the Benjamini-Hochberg (BH) method. A BH-adjusted *P* value of <0.05 was considered statistically significant.

### Metagenomic species clustering.

The gene relative abundance profiles of 326 laboratory mice were clustered using the coabundance canopy algorithm (21). Coabundance genomes (CAGs) that were present in >90% samples were chosen, and CAGs with >700 genes were considered metagenomic species (MGSs) (21). CAGs and MGSs were assigned to a given taxon when >50% genes belonged to that specific taxon (21). The taxonomic distribution of MGSs was calculated using the R package phytool (48). The Shannon index was calculated based on the MGS profiles of samples from the 7 selected mouse groups, and PCoA was based on the same profile.

All MGSs were searched against the 830 high-quality metagenome assembly genomes (MAGs) and the 115 mGMB genomes by MUMmer3 (v3.23) (49) for calculation of MUMi values (22, 23). If the MUMi value for two items was >0.54, then these two items were recognized as the same species (23). The result of the comparison between MGSs and MAGs was hierarchically clustered by R package hclust with weighted pair group method with averaging (WPGMA) and then visualized in a cladogram with annotation by a R package ggtree (50, 51). The result of the comparison between MGSs and mGMB genomes is presented in Table S1J.

### Data availability.

The host-free sequenced data and assembled metagenomes of 88 mice in this study have been deposited in the China National GenBank Sequence Archive with project ID CNP0000619. EMGC can be reached by link http://ftp.cngb.org/pub/CNSA/data2/CNP0000619/Other/. The public data sets presented in this study can be found in online repositories. The names of the repositories and accession numbers can be found in Table S1B and S1C.
