# Supplementary figures and images for: An Expanded Gene Catalog of Mouse Gut Metagenomes
Source: mSphere. 2021 Feb 24;6(1):e01119-20. doi: 10.1128/mSphere.01119-20 (PMC8544893; doi:10.1128/mSphere.01119-20)

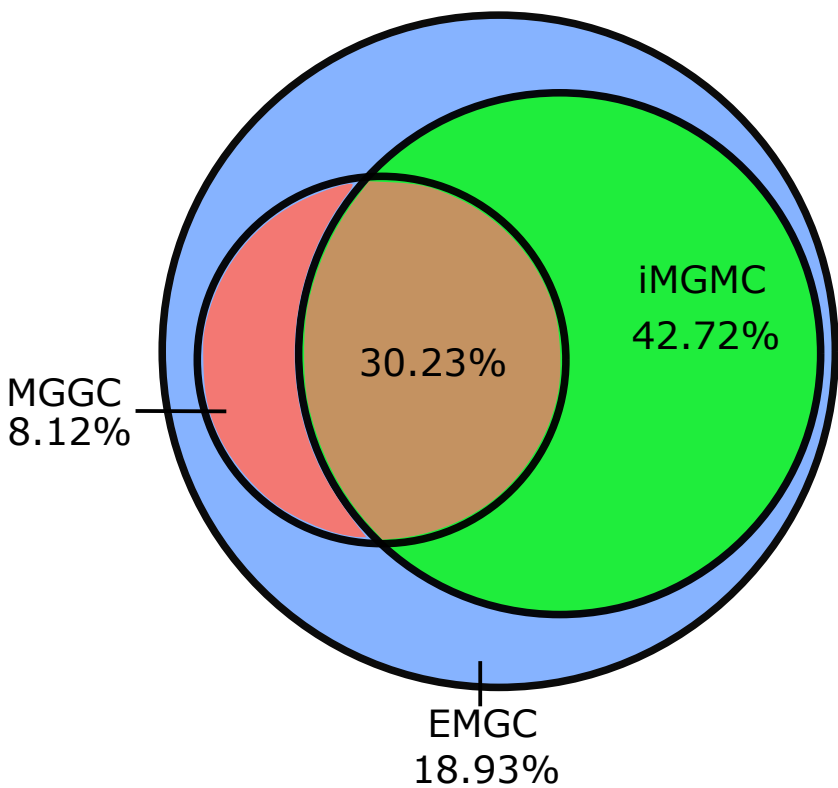

Supplement: FIG S1 [file msphere.01119-20-sf001.pdf]

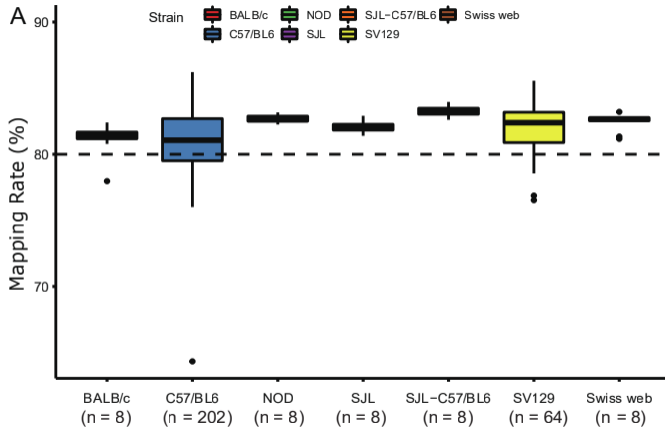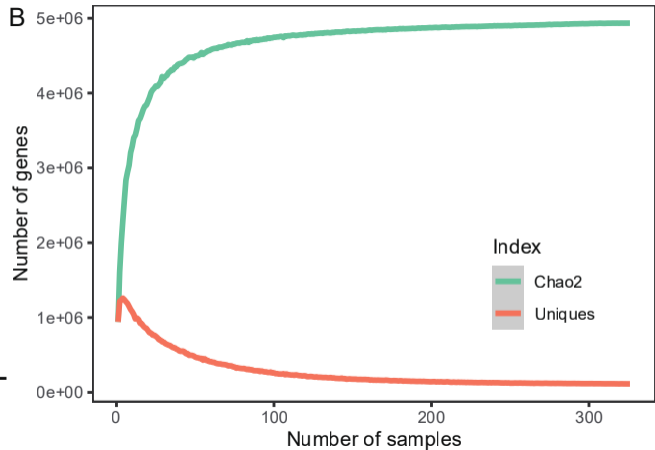

Supplement: FIG S2 [file msphere.01119-20-sf002.pdf]

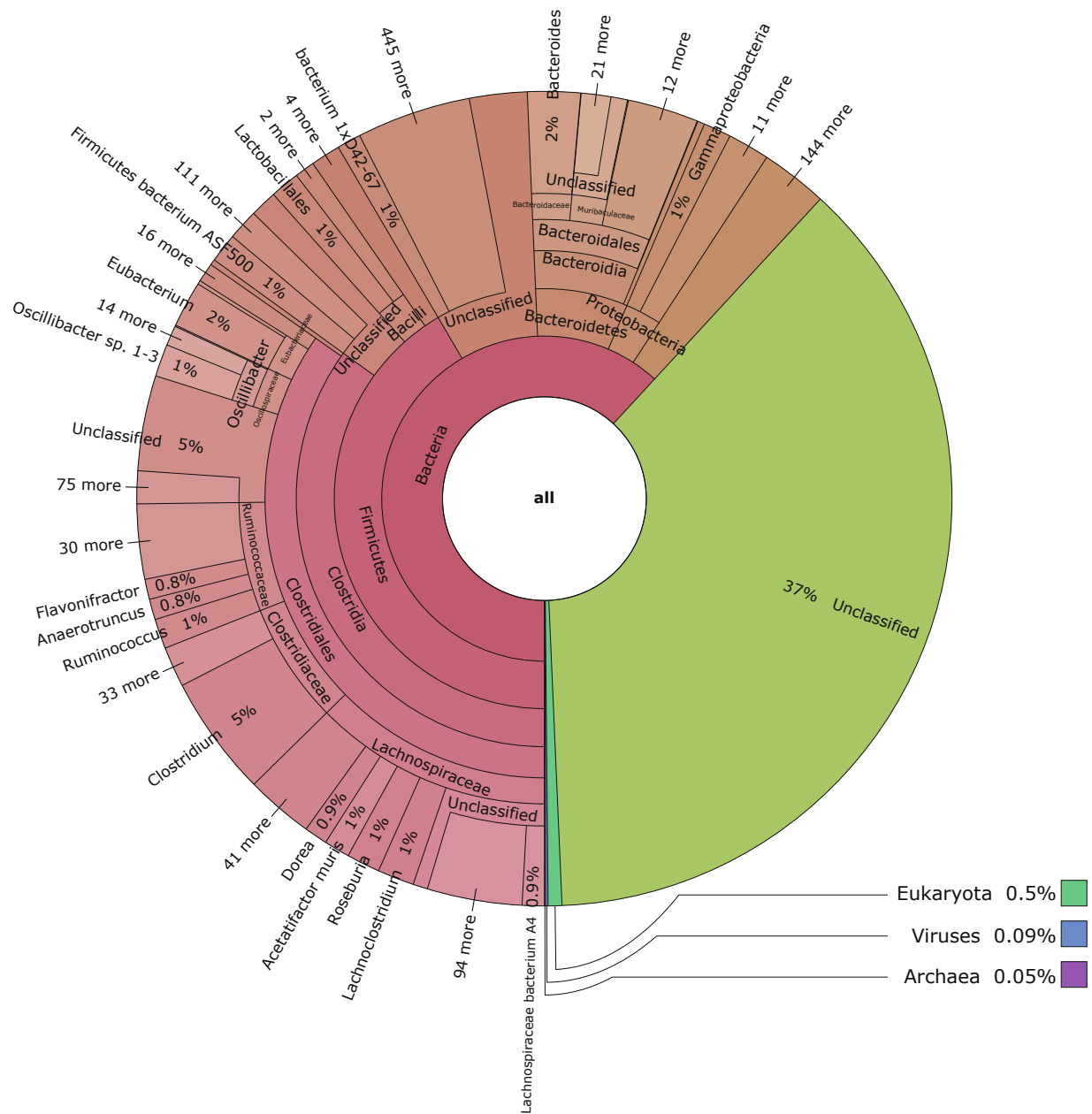

Supplement: FIG S3 [file msphere.01119-20-sf003.pdf]

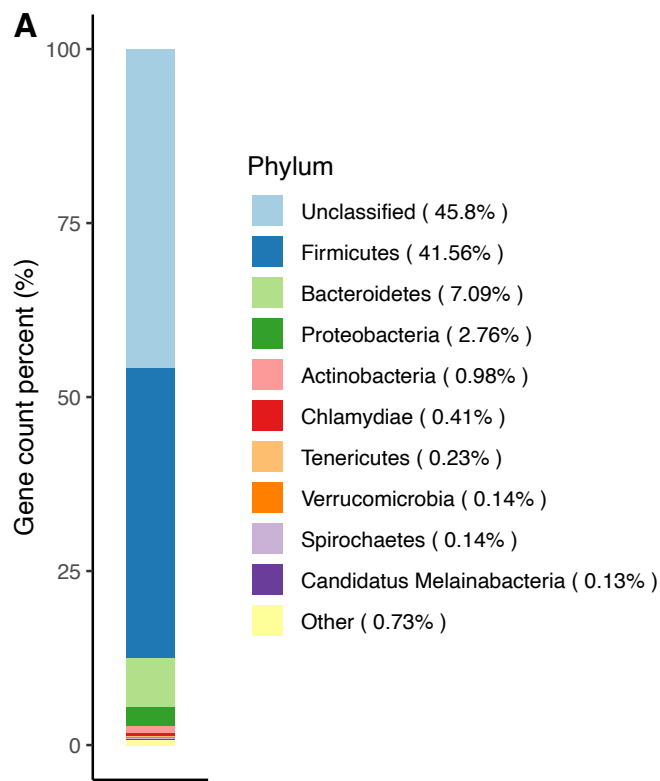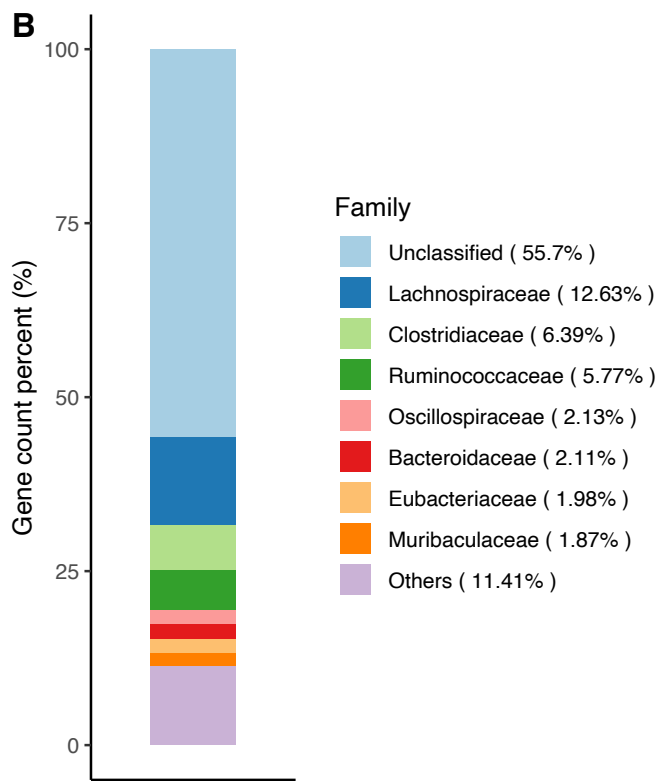

Supplement: FIG S4 [file msphere.01119-20-sf004.pdf]

Second.Level

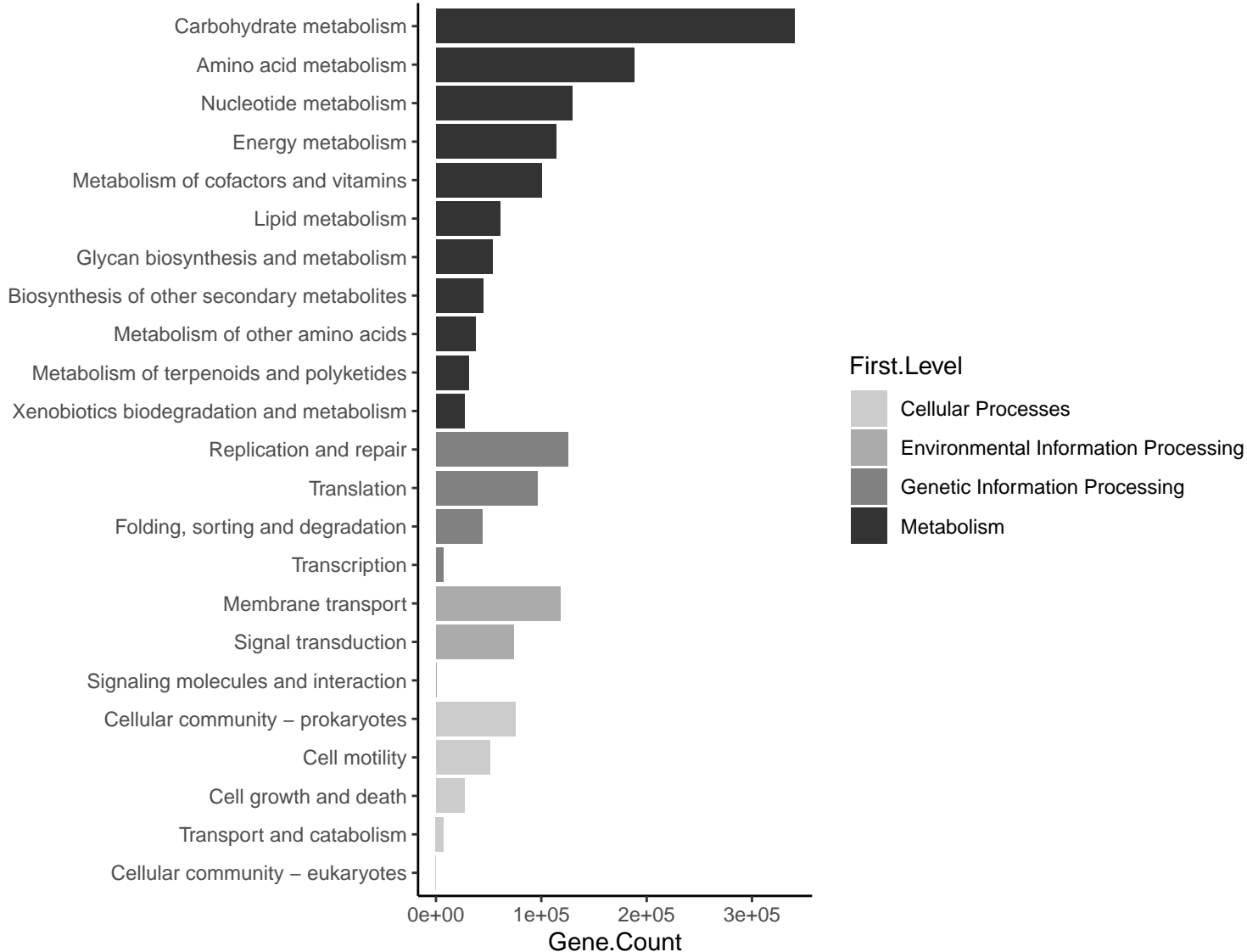

Supplement: FIG S5 [file msphere.01119-20-sf005.pdf]

**A**

Variance of Explained (%)

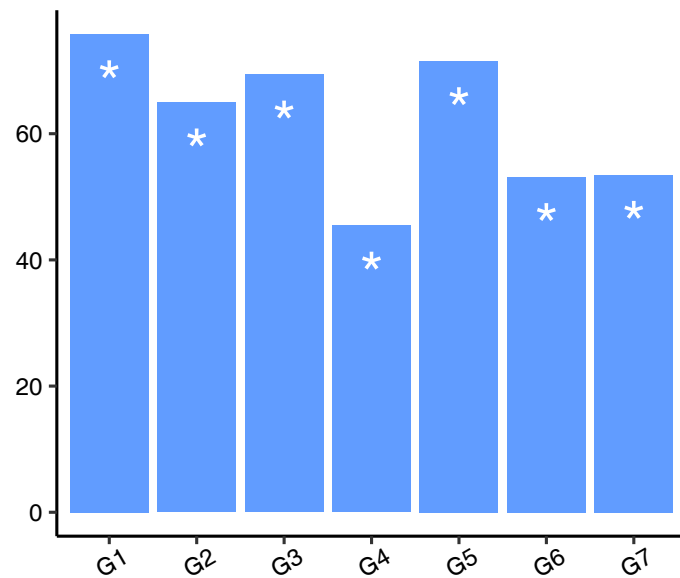**B**

Shannon index

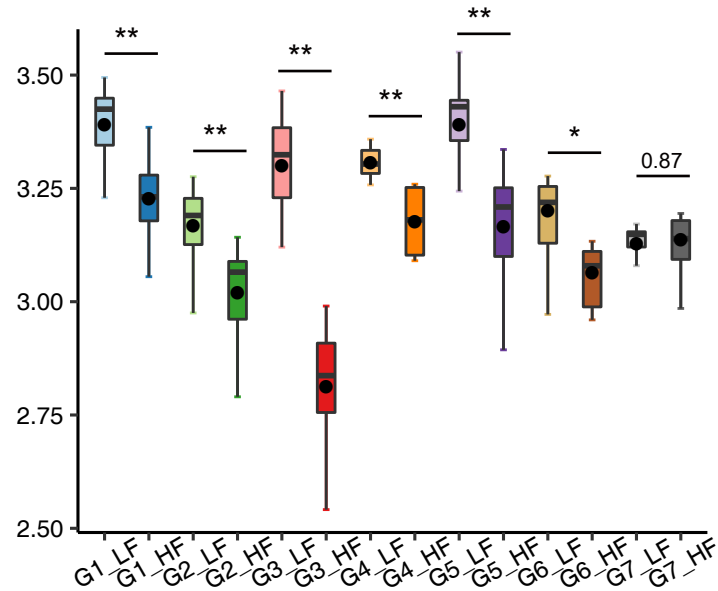**C**

PCoA2 (9.2%)

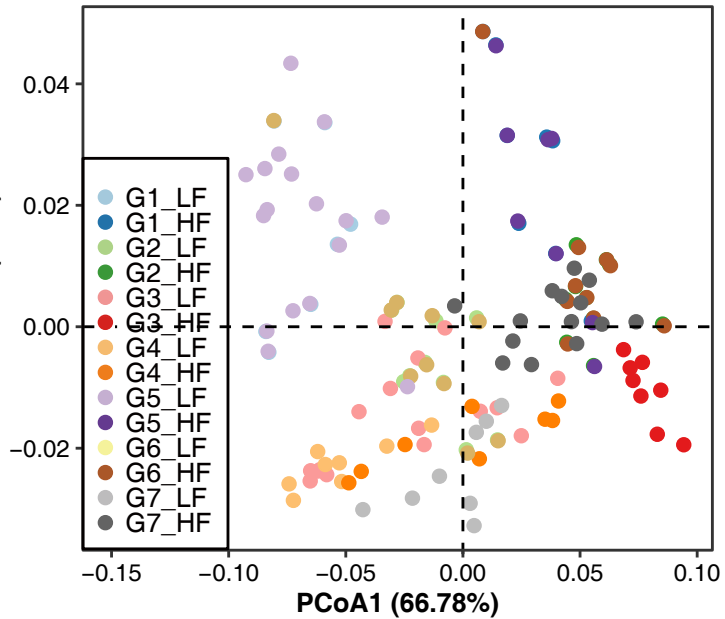

Supplement: FIG S6 [file msphere.01119-20-sf006.pdf]

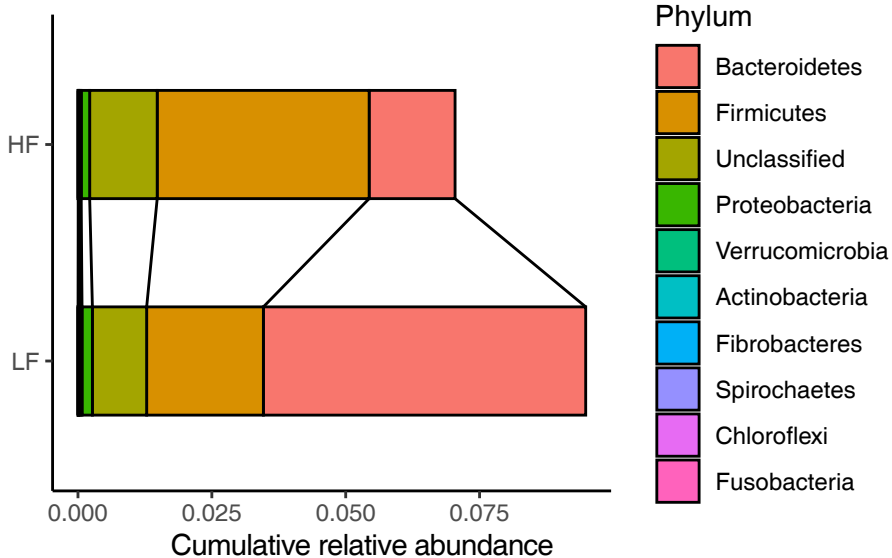

Supplement: FIG S7 [file msphere.01119-20-sf007.pdf]

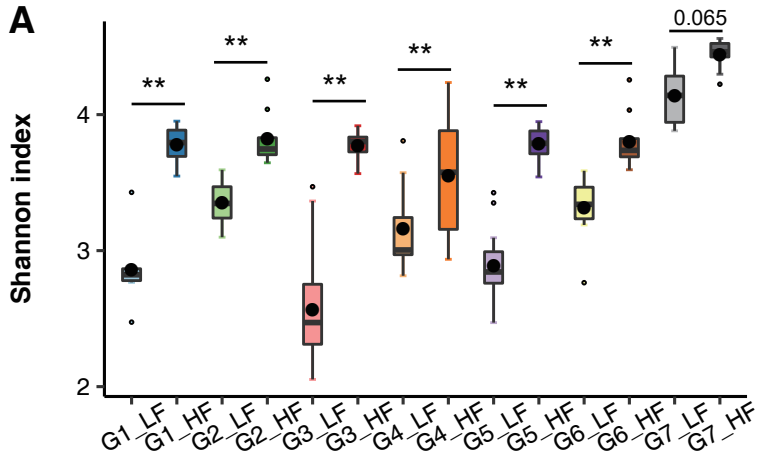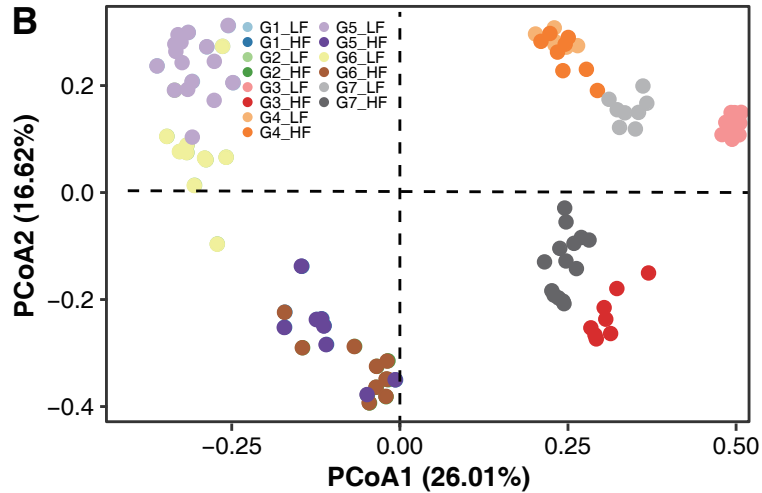

Supplement: FIG S9 [file msphere.01119-20-sf009.pdf]
