# Supplementary material for: An Expanded Gene Catalog of Mouse Gut Metagenomes
Source: mSphere. 2021 Feb 24;6(1):e01119-20. doi: 10.1128/mSphere.01119-20 (PMC8544893; doi:10.1128/mSphere.01119-20)
Supplement: FIG S8 [file msphere.01119-20-sf008.pdf]

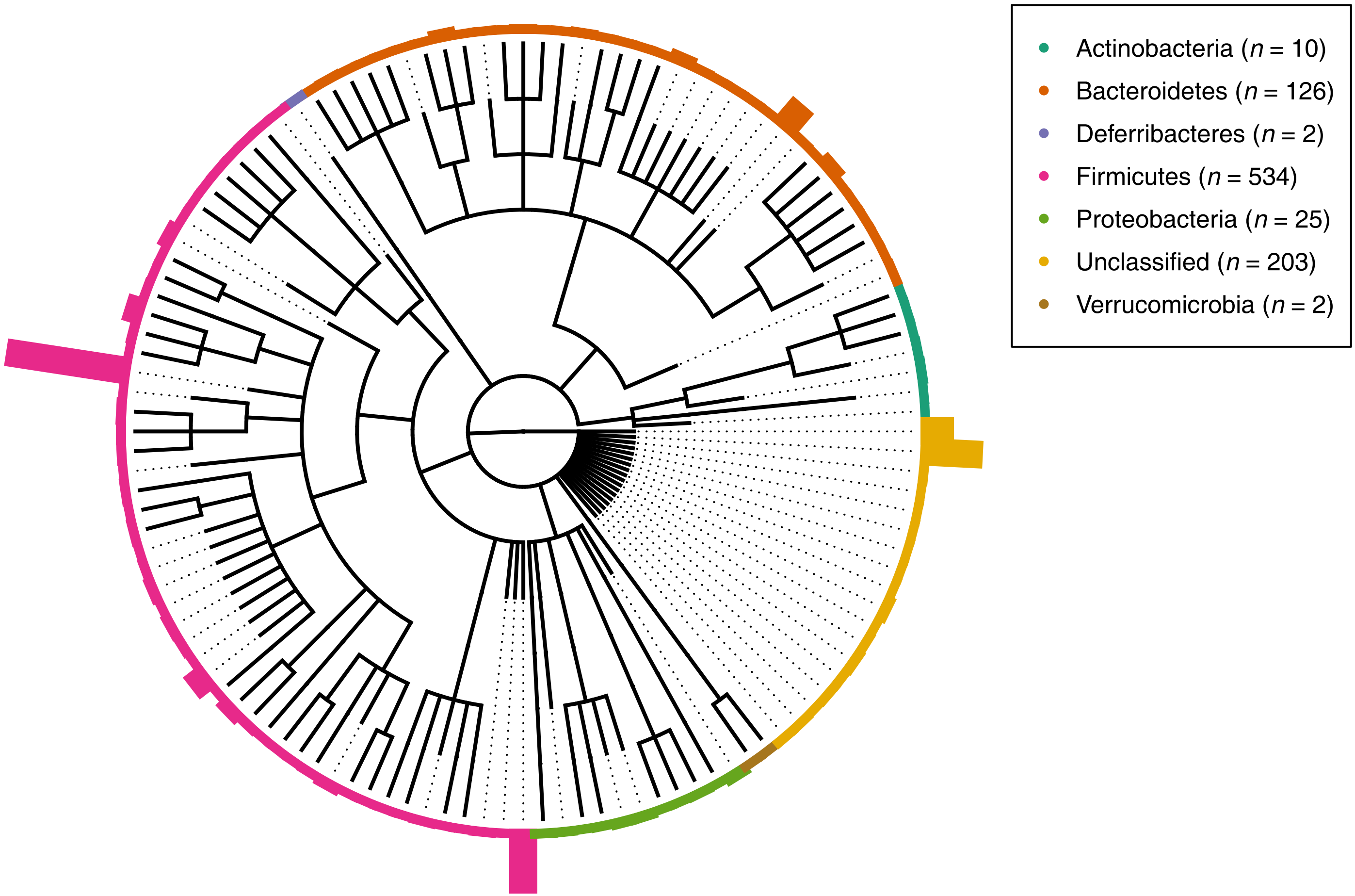

Actinobacteria (*n* = 10)
Bacteroidetes (*n* = 126)
Deferribacteres (*n* = 2)
Firmicutes (*n* = 534)
Proteobacteria (*n* = 25)
Unclassified (*n* = 203)
Verrucomicrobia (*n* = 2)
